# Spousal Caregiving and Cognitive Trajectories: Does Care Recipient Dementia Status Matter?

**DOI:** 10.1093/geroni/igaa057.1157

**Published:** 2020-12-16

**Authors:** Taylor Atkinson, Dylan Jester, William Haley

**Affiliations:** University of South Florida, Tampa, Florida, United States

## Abstract

Caregiving is often considered stressful, even more so if the care recipient has been diagnosed with dementia. The current study examines the rate of cognitive decline of spousal caregivers of persons with dementia (CG-D) when compared to spousal caregivers of persons without dementia (CG) before and after the death of the care recipient. Health and Retirement Study (HRS) data from 1998-2016 were used to examine cognitive trajectories of CG-D (n=364) and CG (n=1,649) before and after the care recipient death. Cognition was measured through the HRS’s shortened Telephone Interview of Cognitive Status and separated into measures of total cognition and memory. Covariates included age, education, sex, race, ethnicity, care hours, frailty, socioeconomic status, nursing home placement of the recipient, and whether the death was expected. Piecewise mixed models were constructed to examine two two-year periods of decline leading up to the death of the care recipient, and two two-year periods of decline after the death of the care recipient. CG-D and CG declined at equivalent rates on measures of total cognition and memory (ps > .05). In all caregivers, total cognition and memory declined at a stable rate before the death of the care recipient. However, an accelerated decline was evident after the death of the care recipient (ps < .001). Our results suggest that cognitive decline is not differentially affected by care recipient dementia diagnosis. We find evidence that the death of a spousal care recipient is accompanied by hastened cognitive decline in our population-based sample.

